# BCL-11 enables adaptive stress responses to environmental challenges

**DOI:** 10.1016/j.isci.2025.114422

**Published:** 2026-01-07

**Authors:** Patrizia Niedworok, Rossella Erminia Ciliberti, Beijia Xie, Amal John Mathew, Benjamin Jussila, Jennifer A. Lawson, Elena De Domenico, Stefan Paulusch, Marc Beyer, Pierluigi Nicotera, N. Ahmad Aziz, Dan Ehninger, Sandra Blaess, Daniele Bano

**Affiliations:** 1 German Center for Neurodegenerative Diseases (DZNE), 53127 Bonn, Germany; 2 University of Bonn, University Hospital Bonn, Institute of Reconstructive Neurobiology, 53127 Bonn, Germany; 3 InVivo Biosystems, Eugene, OR 97402, USA; 4 PRECISE Platform for Single Cell Genomics and Epigenomics, DZNE and University of Bonn and West German Genome Center, 53127 Bonn, Germany; 5 IRCCS Mondino Foundation, via Mondino 2, 27100 Pavia, Italy; 6 Institute for Medical Biometry, Informatics and Epidemiology (IMBIE), Faculty of Medicine, University of Bonn, 53127 Bonn, Germany; 7 Department of Neurology, Faculty of Medicine, University of Bonn, 53127 Bonn, Germany

**Keywords:** health sciences, biological sciences, molecular biology, cell biology

## Abstract

Insulin/IGF-1 signaling (IIS) is a master regulator of metabolism, stress resilience, and cell homeostasis in multicellular organisms. In the nematode *Caenorhabditis elegans*, DAF-2 regulates dauer diapause, animal growth, and lifespan extension in a DAF-16/FOXO-dependent manner. Here we investigated IIS in animals expressing pathogenic variants of BCL-11, an evolutionarily conserved transcription factor that has been implicated in human neurodevelopmental disorders. We found that hypomorphic *bcl-11* mutations have a limited impact on *C*. *elegans* growth and survival under standard growth conditions. On the contrary, BCL-11 deficiency compromises the cytoprotective properties of *daf-2* signaling upon animal exposure to stress. During embryonic development, *daf-16* loss of function rescues egg hatching defects in *daf-2;bcl-11* mutants, suggesting a transcriptional interplay between BCL-11 and DAF-16 in IIS-deficient animals. Together, our data suggest that BCL-11 actively regulates transcription during development, while in adult animals it is recruited in response to environmental insults to enhance stress resilience.

## Highlights

• Hypomorphic *bcl-11* mutants do not show obvious phenotypes
• *daf-2;bcl-11* mutants display altered transcriptional profiles under stress
• DAF-16 loss rescues developmental defects in *daf-2;bcl-11*-mutant animals
• BCL-11 deficiency impairs the cytoprotective properties of *daf-2* signaling

## Graphical abstract

**Figure:**
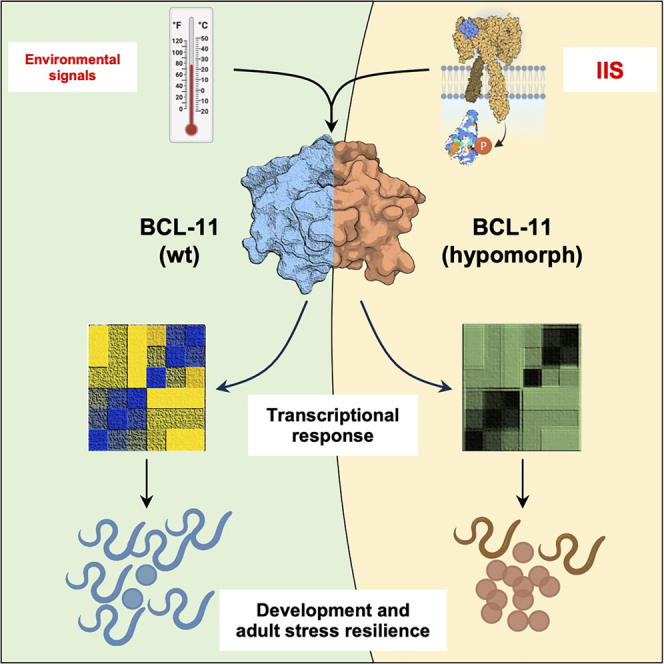

## Introduction

Insulin and insulin-like growth factor 1 (IGF-1) signaling (IIS) plays a critical role in neuronal differentiation and maintenance, since it regulates proteostasis (protein synthesis and degradation) and metabolism.^1^ Upon binding of insulin and/or IGF-1 to their respective receptors, phosphatidylinositol 3-kinase (PI3K) activates serine/threonine kinase AKT, which promotes posttranslational modifications of distinct transcription factors (e.g., FOXO) regulating the expression of genes necessary to support cell growth and survival. As the main metabolic switch in metazoans, IIS controls the use of nutrients according to the environmental conditions to which animals are exposed, thereby modulating the investment of resources that are required to sustain stress resilience and lifespan-extending programs.^2^^,^^3^^,^^4^ In this regard, inhibition of the insulin/IGF-1/DAF-2 signaling doubles the lifespan of the nematode *Caenorhabditis elegans* by promoting the expression of thousands of genes under the control of an array of transcription factors, including DAF-16/FOXO.^5^^,^^6^^,^^7^^,^^8^ While IIS contribution to longevity is well-established, mechanistic aspects on animal development and stress resilience require further investigation.

The B-cell leukemia/lymphoma 11 (BCL11)-family members are highly conserved Krüppel-like C2H2 zinc finger (Znf) transcription factors^9^ that have been described as associated factors of the ATP-dependent chromatin remodeling SWI/SNF/BAF complex.^10^ For both human *BCL11A* and *BCL11B* genes, alternative splicing gives rise to short and long isoforms with variable numbers of Znf domains.^11^^,^^12^ While the N-terminal motif is involved in the homo- and hetero-dimerization,^13^ the canonical C-terminal C2H2 Znf domains are necessary for sequence-specific DNA binding to target genes. Moreover, these Znf sequences significantly contribute to the binding affinity to the DNA duplex and define the levels of transcriptional repression and/or activation of targeted genes.^11^^,^^12^ BCL11-family members were initially identified as chromosomal translocations or amplifications in lymphoid malignancies.^14^ However, later clinical evidence reported their contribution in regulating the switch from fetal to adult hemoglobin expression, further supporting their involvement in inherited forms of blood disorders (e.g., beta-thalassemia, sickle cell disease).^15^^,^^16^^,^^17^^,^^18^ Moreover, pathogenic BCL11A and BCL11B variants have been implicated in rare forms of neurodevelopmental disorders (NDDs) with variable degrees of intellectual disability, behavioral abnormalities and other tissue-specific traits (e.g., scoliosis, immune system dysfunction).^19^^,^^20^^,^^21^ In addition to the growing body of genetic evidence implicating BCL11A and BCL11B involvement in NDDs, multiple studies have demonstrated that BCL11 proteins contribute to embryonic brain development and adult neurogenesis in experimental model organisms.^11^^,^^22^ For example, *Bcl11b* knockout alters the axonal growth of spinal cord neurons and impairs hippocampal neurogenesis in rodent animal models,^23^^,^^24^ whereas forebrain-specific *Bcl11a* knockout induces neurodegeneration in the somatosensory cortex by directly activating the transcription factor BCL6 that elicits DNA damage response.^25^ Inactivation of both *Bcl11a* and *Bcl11b* in the developing cortex results in aberrant proliferation and premature differentiation of cortical progenitor cells.^26^ Notably, prenatal *Bcl11a* knockout in midbrain dopaminergic neurons increases their susceptibility to oxidative stress and alpha-synuclein overexpression in adulthood.^27^ Taken together, these experimental data suggest that loss of BCL11 proteins leads to massive changes in neuronal differentiation, maintenance and survival, providing insights into the potential mechanisms that could cause BCL11-associated NDDs. However, while the downstream phenotypic consequences of BCL11A/B deficiency have been described, less is known about the signaling cascades and transcriptional activities that depend on BCL11 proteins. Recently, a prospective study in three independent cohorts has reported that western-style diets during pregnancy are associated with NDDs, further highlighting the importance of metabolic insults on prenatal developmental programs.^28^ Consistently, IGF-1 deficiency in perinatal mice may cause behavioral alterations that resemble autistic traits in children born preterm.^29^ Notably, mouse phospho-proteome data have shown a clear enrichment of proteins associated with neuropsychiatric disorders, including BCL11A.^29^ Motivated by this potential link between IIS and BCL11-family members, we decided to test the role of BCL-11 in long-lived *daf-2(e1370)*-mutant *C*. *elegans* strains. We generated new mutant alleles for the sole *bcl-11* homolog gene and studied phenotypes at standard growth conditions as well as upon stress. While hypomorphic *bcl-11* mutations did not affect *daf-2* longevity programs at optimal growth temperature, they undermined the development and survival of *daf-2*-mutant nematodes when animals were exposed to environmental challenges in the form of heat stress. Mechanistically, our findings show that BCL-11 can contribute to IIS, since it regulates gene expression profiles during embryogenesis. Additionally, we found that BCL-11 is recruited “on-demand” in adult animals to promote IIS response to environmental challenges, suggesting a novel function of BCL11 proteins in the fine-tuning of transcriptional programs beyond animal development.

## Results

### Modeling disease-causing *BCL11* mutations identifies functionally conserved domains

**Figure 1:**
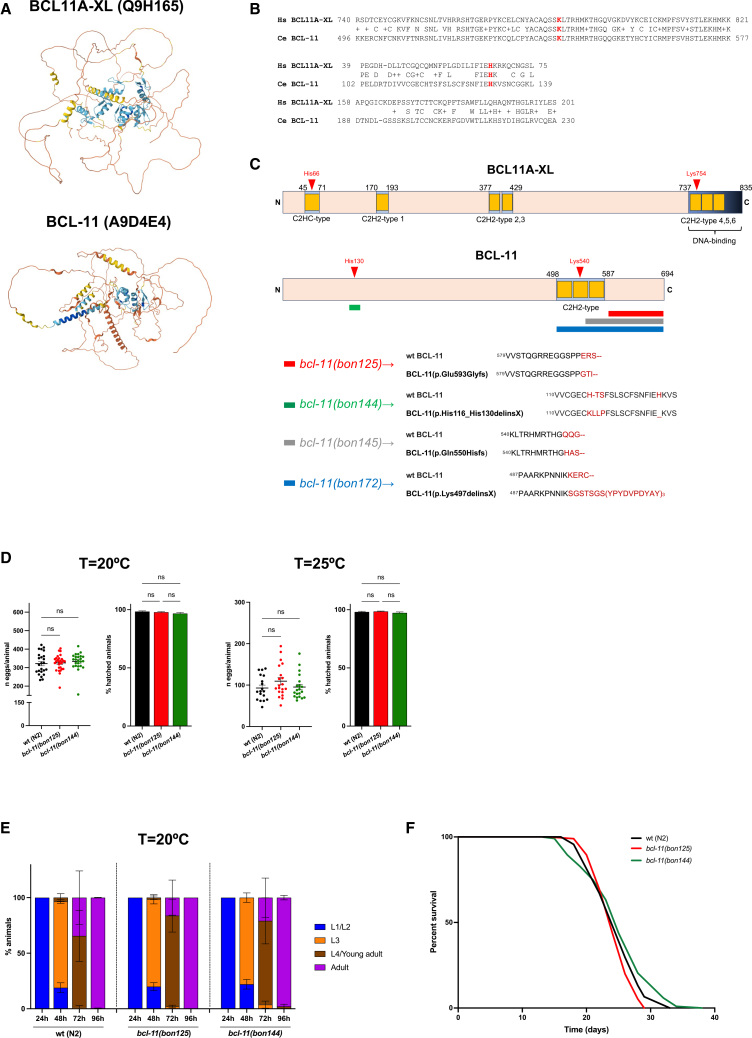
Generation of *bcl-11*-mutant *C*. *elegans* (A) Alphafold structure prediction of human BCL11A-XL (UniProt ID/AC Q9H165) and *C*. *elegans* BCL-11 (UniProt ID/AC A9D4E4). (B) Sequence alignment of human (Hs) BCL11A-XL and *C*. *elegans* (Ce) BCL-11 proteins. Disease-associated residues (in human, histidine 66 and lysine 754) are highlighted in red letters. (C) Schematic representation of the structural domains in BCL11A-XL and BCL-11, as annotated in UniProt. Zing finger motifs (C2HC-type and C2H2-type) and disease-associated residues (in red) are indicated. Color-coded rectangles mark CRISPR-Cas9-generated mutations. At the bottom, predicted amino acid sequences for each mutant allele, derived from DNA sequencing data, are shown. Alignments highlight predicted deletions and insertions (in dark red). (D) Egg-laying and hatching assay. Total number of laid eggs (left) and percentage of hatched animals (right) at the indicated temperatures (number of animals = 18–28 from three independent experiments pooled together; one-way ANOVA with Dunnett’s multiple comparisons test, ns = not significant). (E) Developmental assay of wt, *bcl-11(bon125)-* and *bcl-11(bon144)*-mutant animals at 20°C (mean ± SEM, number of animals = 65–130 from three independent experiments). (F) Representative lifespan assays of wt, *bcl-11(bon125)*, and *bcl-11(bon144) C*. *elegans* at 20°C.

BCL11-family members harbor multiple Znf domains (seven in BCL11A-XL) and extensive intrinsically disordered regions (Figure 1A), which provide structural flexibility and facilitate dynamic interactions with DNA as well as chromatin remodeling complexes.^30^^,^^31^ Given the presence of evolutionarily conserved motifs (Figure 1B), we designed gene editing strategies to generate hypomorphic and recessive *loss-of-function* *(lof)* alleles of the *bcl-11/F13H6*.*1* gene, thereby obtaining BCL-11 variants that partially resemble mutations previously identified in NDD patients.^21^^,^^32^ We isolated four *bcl-11*-mutant alleles, one of which (*bon172*) encoded a truncated BCL-11 lacking ∼196 amino acids and carrying a predicted triple HA sequence (3xHA) at the C terminus (Figure 1C). A second allele (*bon145*) was a predicted frameshift that caused the loss of at least one C2H2-type domain and possibly resulted in a premature stop codon (Figure 1C). Both alleles were *lof* and were maintained with the *nT1* balancer, a stable reciprocal translocation to maintain lethal or sterile mutations at chromosomes IV and V. When we tried to retrieve homozygous *bcl-11(bon172)* mutant animals, we were unable to isolate adult hermaphrodites, although we did observe a very small number (<1%) of *bcl-11* homozygous mutant larvae that failed to develop further. Of note, similar phenotypes were observed in animals expressing the *bcl-11(bon145)* allele. While these two *lof* alleles (*bon145* and *bon172*) caused developmental defects, the other two *bcl-11* mutant *C*. *elegans* strains were viable and could be maintained in homozygosity. We sequenced *bcl-11* (*bon125*) allele and found a frameshift mutation that resulted in a variant with intact C2H2-type Znf domains, although with an insertion of ∼64 amino acids after proline 592 (Figure 1C). Instead, *bcl-11(bon144)* was an in-frame deletion-insertion that affected an evolutionarily conserved domain associated with NDDs^32^ (Figure 1C). After backcrossing, neither mutation affected adult egg laying, larval hatching or development (Figures 1D and 1E). Under standard experimental growth conditions, neither *bcl-11(bon125)* nor *bcl-11(bon144*) showed obvious lifespan differences compared to wild-type (wt) animals (Figure 1F and Table S1). Together, these data suggest that hypomorphic *bcl-11* mutations do not induce any obvious phenotypes.

### Hypomorphic BCL-11 variants have limited influence in *C*. *elegans* adults

**Figure 2:**
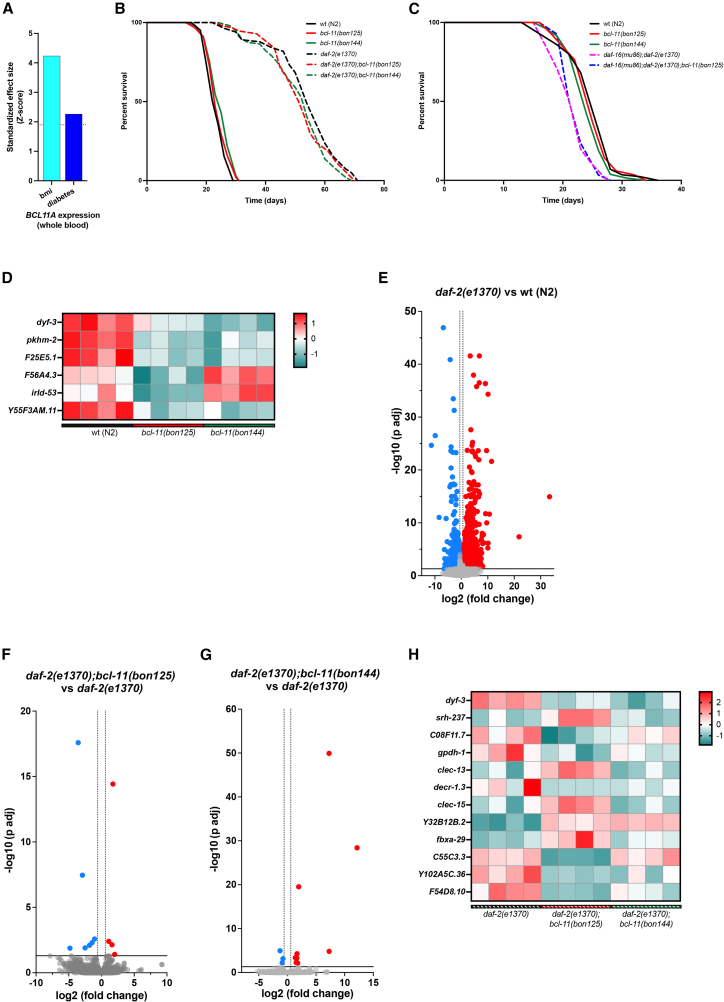
Hypomorphic BCL-11 variants do not affect animal survival under standard conditions (A) Genetically determined expression levels of *BCL11A* in whole blood were significantly associated with both a higher body mass index and a higher risk of type 2 diabetes. The horizontal dashed line represents the level of statistical significance (i.e., standardized effect size, *Z* > 1.96, *p* value <0.05). (B) Lifespan assay of wt and mutant nematodes at 20°C. The genotypes of the six strains are reported next to the graph. (C) Representative lifespan assay of *daf-16(mu86);daf-2(e1370)* and *daf-16(mu86);daf-2(e1370)*;*bcl-11(bon125)* animals compared to wt and *bcl-11* single mutant nematodes at 20°C. (D) Heatmap of commonly dysregulated genes in *bcl-11(bon125)* and *bcl-11(bon144)* mutants compared to wt. Gene expression values were log-transformed and standardized per gene (row-scaled) to obtain *Z* scores. The color gradient indicates relative expression levels for each gene: red and green represent higher expression (*Z* > 0) and lower expression (*Z* < 0), respectively. (E) Volcano plot of *daf-2(e1370)* mutants compared to wt animals at 20°C; threshold: abs (log2 (fold change)) > 0.585, p adj <0.05. (F–G) Volcano plots of (F) *daf-2(e1370);bcl-11(bon125)* vs. *daf-2(e1370)* and (G) *daf-2(e1370);bcl-11(bon144)* vs. *daf-2(e1370)* animals at 20°C; threshold: abs (log2 (fold change) > 0.585, p adj <0.05). (H) Heatmap of the 12 dysregulated genes identified in both *daf-2(e1370);bcl-11(bon125)* and *daf-2(e1370);bcl-11(bon144)* compared to *daf-2(e1370)* animals. Gene expression values were row-scaled to obtain *Z* scores. As in (D), the color gradient represents relative expression levels for each gene (red = higher expression; green = lower expression).

Recent evidence has revealed that IGF-1 receptor inhibition causes BCL11A hyperphosphorylation in newborn mouse pups.^29^ By combining data from expression quantitative trait loci (eQTLs) in whole blood from the Genotype-Tissue Expression project with data from genome-wide association studies (GWASs) on body mass index (BMI) and type 2 diabetes, we found a clear association between a higher *BCL11A* expression and both a higher BMI and a higher risk of type-2 diabetes in humans (Figure 2A). Moreover, leveraging the ExPheWas platform to conduct gene-based phenome-wide association studies,^33^ we identified a large number of metabolic traits to be significantly associated with genetic polymorphisms in the *BCL11A* gene in humans, including BMI, diabetes, body fat percentage, waist circumference, basal metabolic rate, as well as levels of glycated hemoglobin (HbA1c) and HDL cholesterol (all FDR-adjusted *p* values <0.05) (Table S2). Inspired by the potential link between metabolism in IIS and BCL11 family members, we set out a series of experiments in *daf-2*-mutant *C*. *elegans*. Contrary to our expectation, hypomorphic *bcl-11* mutations affected neither the lifespan extension of *daf-2* mutants nor the survival of *daf-16;daf-2*-mutant animals (Figures 2B and 2C; Table S1). Since BCL11-family members are critical regulators of gene transcription,^11^ we sought to test the impact of *bcl-11* mutations on *C*. *elegans* transcriptional profiles. To do so, we collected young adult nematodes grown at 20°C and carried out RNA-sequencing analysis. Compared to wt animals, *bcl-11(bon125)* and *bcl-11(bon144)* mutants showed 155 and eight dysregulated genes, respectively, six of which were common between the two hypomorphic *bcl-11* mutants (Figure 2D). We went on and assessed the transcriptional profiles of *daf-2* mutants, since IIS inhibition promotes significant changes in gene expression (Figure 2E). Contrary to our expectations, the number of dysregulated genes in *daf-2;bcl-11* double mutants was relatively small (Figures 2F and 2G), with 12 shared genes that were commonly dysregulated in *daf-2(e1370);bcl-11(bon144)* and *daf-2(e1370);bcl-11(bon125)* animals (Figure 2H). Together, these data suggest that hypomorphic BCL-11 mutations have a limited impact on IIS in animals grown under standard experimental conditions.

### BCL-11 is required to sustain stress resilience in *daf-2*-mutant nematodes

**Figure 3:**
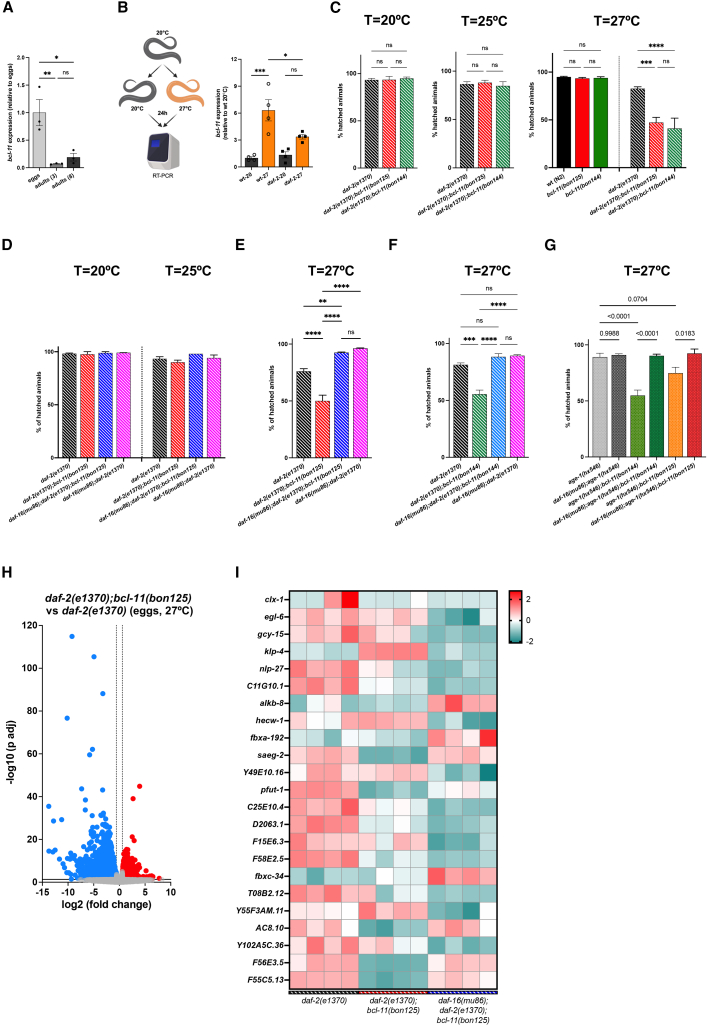
Hypomorphic BCL-11 variants negatively affect *daf-2* development at warmer temperatures (A) Representative RT-PCR analysis of *bcl-11* expression in eggs and adult (day 3 and 8, after hatching) nematodes at 20°C (*n* biological replicates = 3; mean ± SEM, one-way ANOVA with Tukey’s multiple comparisons test, ns = not significant, *∗p* < 0.05, *∗∗p* < 0.01). (B) Schematic representation of the experimental design (left) was created in BioRender. Bano, D. (2026) https://BioRender.com/wczv7ej. At the right, RT-PCR of *bcl-11* expression in wt and *daf-2(e1370)*-mutant animals. Samples are from animals constantly maintained at 20°C (dark gray columns), or grown at 20°C and then shifted at 27°C for 24 h (orange bars) (*n* biological replicates = 4; mean ± SEM, one-way ANOVA with Tukey’s multiple comparisons test, ns = not significant, *∗p* < 0.05, *∗∗∗p* < 0.001). (C) Percentage of viable animals that hatched after 48 h incubation at the indicated temperatures (*n* biological replicates >5; mean ± SEM, one-way ANOVA with Šidák’s multiple comparisons test, ns = not significant, *∗∗∗p* < 0.001, *∗∗∗∗p* < 0.0001). (D–E) Hatching rate assay at the indicted temperatures (for D, *n* = 2; for E, *n* = 6, including double-blinded exp; mean ± SEM, one-way ANOVA with Tukey’s multiple comparisons test, ns = not significant, *∗∗p* < 0.01, *∗∗∗∗p* < 0.0001). (F) Hatching rate assay of *daf-2(e1370)*, *daf-2(e1370);bcl-11(bon144)*, *daf-16(mu86);daf-2(e1370);bcl-11(bon144)* and *daf-16(mu86);daf-2(e1370)* at 27°C (*n* = 3, one-way ANOVA with Tukey’s multiple comparisons test, ns = not significant, *∗∗∗p* < 0.001, *∗∗∗∗p* < 0.0001). (G) Hatching rate assay at 27°C of *age-1(hx546)*, *daf-16(mu86);age-1(hx546)*, *age-1(hx546);bcl-11(bon144)*, *daf-16(mu86);age-1(hx546);bcl-11(bon144)*, *age-1(hx546);bcl-11(bon125)*, and *daf-16(mu86);age-1(hx546);bcl-11(bon125)* (*n* = 4, one-way ANOVA with Tukey’s multiple comparisons test: *p* values are reported in the graphs). (H) Volcano plot of dysregulated genes in *daf-2(e1370);bcl-11(bon125)* vs. *daf-2(e1370)* embryos. Eggs were exposed to 27°C and RNA was extracted after 2 h threshold: abs (log2 (fold change)) > 0.585, p adj <0.05. (I) Heatmap of differentially expressed genes identified in *daf-16(mu86);daf-2(e1370);bcl-11(bon125)* vs. *daf-2(e1370);bcl-11(bon125)*, showing their expression profiles across all samples. RNA was extracted from eggs incubated at 27°C for 2 h. Gene expression levels were log-transformed and then scaled by row (per gene) to obtain *Z* scores. Color codes indicate expression levels (red = upregulation; green = downregulation).

We carried out RT-PCR and observed that *bcl-11* expression peaked during embryonic development, while it was significantly downregulated at adulthood (Figure 3A). Surprisingly, *bcl-11* gene expression was significantly upregulated when adult animals were shifted from 20°C to 27°C for 24 h (Figure 3B), suggesting that *bcl-11* may be a stress-induced gene. Thus, we tested the impact of hypomorphic *bcl-11* mutations in *daf-2*-mutant embryos at standard growth temperature (20°C) as well as at warmer conditions, knowing that high temperatures (>25°C) represent adverse environmental stimuli that can profoundly affect *C*. *elegans* physiology.^34^^,^^35^ While hypomorphic *bcl-11* mutations had no effects on egg hatching at 20°C and 25°C, they significantly reduced the hatching and survival of *daf-*2 embryos when exposed to 27°C (Figure 3C). Since DAF-16 is a master regulator of DAF-2 stress resilience,^6^^,^^7^^,^^36^^,^^37^ we generated *daf-16;daf-2;bcl-11* triple mutants and found that DAF-16 loss could completely rescue the hatching defects of *daf-2* mutants carrying a hypomorphic *bcl-11* mutation (Figures 3D–3F), underscoring a possible transcriptional interplay between BCL-11 and DAF-16 in IIS-deficient animals. Consistently, we observed a similar effect in *bcl-11* mutants expressing the hypomorphic *age-1(hx546)* allele (Figure 3G), a genetic mutation that inhibits AGE-1/PI3K activity and promotes lifespan extension.^38^

To profile transcriptional changes upon stress, we extracted eggs from *daf-2*, *daf-2;bcl-11* and *daf-16;daf-2;bcl-11* mutant animals grown at 20°C. Then, we incubated the eggs at 27°C for 2 h and carried out RNA-sequencing analysis. Contrary to what we observed in adult nematodes grown at 20°C (Figures 2F–2H), thousands of genes were dysregulated in *daf-2;bcl-11* double mutants compared to *daf-2*-mutant animals when exposed to a warmer temperature (27°C) (Figure 3H). Of the 23 genes that were altered in both *daf-2;bcl-11* and *daf-16;daf-2;bcl-11* mutants compared to *daf-2* animals (Figure 3I), six genes (*klp-*4, *hecw-1*, *saeg-2*, *AC8*.*10*, *F56E3*.*5*, F55C5.13) were transcriptionally rescued by *daf-16(lof)* to the same levels detected in *daf-2* mutants (Figure 3I).

**Figure 4:**
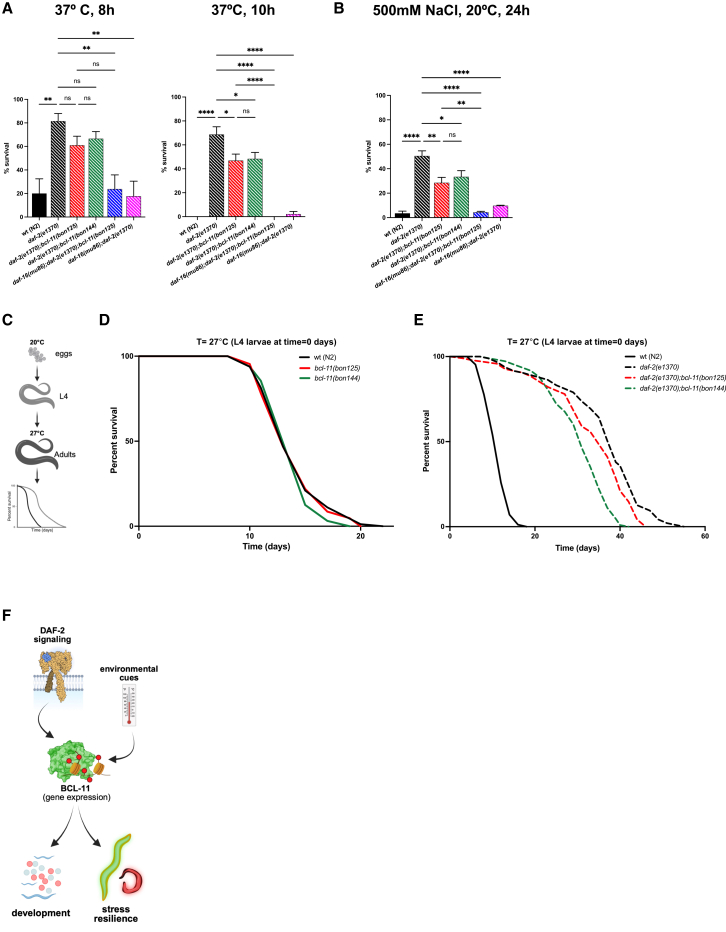
BCL-11 contributes to stress resilience and longevity of adult *daf-2* mutants (A) Heat stress assay. Animals were grown at 20°C and, when at L4 larval stage, transferred to 37°C until all wt animals were dead. After 10 h, *daf-2;bcl-11* double mutants showed reduced heat resistance compared to *daf-2* mutants (*n* = 4; mean ± SEM, ordinary one-way ANOVA with Tukey’s multiple comparisons test, ns = not significant, *∗p* < 0.05, *∗∗p* < 0.01, ∗∗∗*∗p* < 0.0001). (B) High-salt stress assay at 20°C. L4 animals were transferred onto 500 mM NaCl-containing plates and dead animals were scored 24 h after (*n* = 4; mean ± SEM, ordinary one-way ANOVA with Tukey’s multiple comparisons test, ns = not significant, *∗p* < 0.05, *∗∗p* < 0.01, ∗∗∗*∗p* < 0.0001). (C) Schematic representation of the experimental paradigm. Created in BioRender. Bano, D. (2026) https://BioRender.com/wczv7ej. Eggs were extracted from gravid adult animals and seeded on bacteria-containing NGM plates at 20°C. When animals reached L4 larval stage, they were transferred on new plates at 27°C and lifespan assays were carried out. (D–E) Representative lifespan assays of wt and mutant nematodes at the indicated conditions. The genotypes of each strain are shown in the respective graphs. (F) Schematic summary of our findings. BCL-11 integrates IIS and environmental cues to promote animal development and adult stress resilience. Created in BioRender. Bano, D. (2026) https://BioRender.com/wczv7ej.

Having established the impact of hypomorphic BCL-11 variants during development, we extended our investigation to adult nematodes that were subjected to environmental challenges. We performed heat stress and osmotic stress resistance assays and found that hypomorphic *bcl-11* mutations hindered the thermotolerance and cytoprotective effects of IIS-deficient mutants (Figures 4A and 4B). Of note, loss of DAF-16 did not rescue the phenotypes of *daf-2;bcl-11* mutants (Figures 4A and 4B), contrary to what we observed in hatching *daf-2* embryos (Figures 3E and 3F). To further confirm the link between BCL-11 and IIS, we adopted an additional experimental paradigm in which animals were initially grown at 20°C until L4 larval stage to avoid dauer entry, and then transferred to 27°C to measure their survival (Figure 4C). While *bcl-11* single mutants had the same lifespan of wt animals (Figure 4D and Table S1), at least one hypomorphic *bcl-11* mutation appeared to affect the median lifespan of *daf-2(e1370)* (Figure 4E and Table S1), possibly indicating that BCL-11 may contribute to *daf-2*-dependent processes associated with longevity. Together, these data suggest that IIS may recruit BCL-11 “on-demand” to promote stress resilience in response to environmental insults (Figure 4F).

## Discussion

NDDs are a broad and heterogeneous group of conditions that typically become evident in childhood or early adolescence. NDDs have distinct clinical profiles and include autism spectrum disorder, attention-deficit hyperactivity disorder, Tourette syndrome, intellectual disability, and learning and communications disorders.^39^^,^^40^ The prevalence of NDDs has been steadily increasing over the last two decades, probably because of continuous improvements in diagnostic criteria and neuropsychiatric assessment.^41^ With regard to the causes underlying these heterogeneous disorders, there is a clear association between NDDs and the exposure to prenatal and perinatal maternal or environmental factors, including diets, medications, toxins, infections, allergic reactions or non-transmittable conditions (e.g., maternal asthma).^39^^,^^40^^,^^42^^,^^43^^,^^44^ Furthermore, familial and genome-wide association studies have reported an increasing number of genetic risk factors and rare inherited variants that are causally associated with NDDs. In this regard, a growing body of literature has described the contribution of BCL11A/B to the pathogenesis of human brain disorders.^9^^,^^21^ Apart from the reported cases due to *BCL11A* haploinsufficiency, BCL11-related NDDs are associated with *de novo* missense mutations, splice variants and frameshifts that alter evolutionarily conserved domains (e.g., DNA binding regions) or cause premature stop codons, hence resulting in truncated proteins.^19^^,^^21^^,^^32^^,^^45^ Like many other rare genetic disorders, BCL11-related NDDs show a poor genotype-phenotype correlation, with patients that may develop congenital malformations (including craniofacial features), behavioral abnormalities and neurological manifestations (e.g., seizures)^21^ that vary greatly in their clinical presentation and severity.

To better understand the molecular mechanisms associated with BCL11A/B deficiency, it is important to elucidate the temporal recruitment of BCL11A/B during embryonic programs and the extent to which its aberrant activity can irreversibly modify the functional transcriptional network after birth. Equally important, it would be of great interest to investigate how environmental challenges or hormonal stimulations may engage BCL11A/B to regulate cell differentiation and/or maintenance in postnatal organisms as recently suggested.^29^ To this end, we employed a genetically tractable multicellular model organism and sought to explore the mechanistic contribution of BCL-11 to stress response. Thus, we carried out genetic manipulations in *C*. *elegans* and obtained hypomorphic and *lof bcl-11* mutations that could model pathogenic BCL11 variants previously identified in patients.^21^^,^^32^ We successfully generated deletion and in-frame deletion/insertion mutations within evolutionarily conserved domains of BCL11, with two of these variants lacking a fully functional DNA binding domain and unable to support *C*. *elegans* development. As we could retrieve only a handful of homozygous mutant larvae that did not develop to adult animals, we inferred that homozygous *bcl-11(lof)* mutations cause lethality, which is consistent with the current compendium of disease-causing mutations that includes homozygous or compound heterozygous hypomorphic *BCL11A* mutations.^21^^,^^32^ To study how pathogenic BCL11 variants affect organismal physiology, we focused our work on a truncated BCL-11 variant lacking the C-terminal region and an in-frame deletion/insertion within the N-terminal region, the latter having an amino acid stretch that shares sequence similarity with the C2HC-type Znf motif of BCL11A. Both of these two BCL-11 variants could be maintained in homozygosity, supporting their classification as *bona fide* hypomorphic mutations. At standard growth conditions, our mutant strains did not exhibit any obvious brood-size defects and had a lifespan that was comparable to wild types. Although at first glance these data may appear disappointing, they suggest that subtle changes in BCL-11 activity can be well tolerated and do not lead to discernible phenotypes. Motivated by this hypothesis and the recent link between BCL11A and IIS,^29^ we sought to challenge the system and assessed BCL-11 effects in IIS-deficient animals, with the knowledge that DAF-2 is the main regulator of metabolism and stress resilience in *C*. *elegans*.^5^^,^^6^^,^^46^^,^^47^ While hypomorphic *bcl-11* mutations had neither an impact on survival nor on the transcriptional profiles of *daf-2* mutants at 20°C, they negatively affected hatching at a warmer temperature (27°C). These data suggest that our hypomorphic BCL-11 variants have fully penetrant developmental defects when insulin/IGF-1/DAF-2-mutant embryos are shifted from permissive to restrictive temperatures. If so, it appears that two signals—an environmental insult and a metabolic trigger—are required to unveil the pathogenic features of a hypomorphic BCL-11 mutation. Although we could not pinpoint additional downstream molecular players, our rescue experiment provides the first mechanistic evidence that a fully functional BCL-11 is required to antagonize DAF-16/FOXO during embryonic development in DAF-2 deficient nematodes. In this regard, it is known that DAF-16 is a key stress-responsive transcription factors that allocate resources in response to environmental cues, promoting reproductive success by regulating the germline quality maintenance, oocyte production and embryonic development. In an autonomous (gonads) as well as in non-autonomous (e.g., intestine) manner, DAF-16 can modulate the expression of protective genes that prevent the commitment of germ cells to development in the absence of appropriate nutrition.^48^^,^^49^ The quality control effects of DAF-16 in the reproductive system are separable from its influence on organismal aging.^50^^,^^51^ Based on our data, *daf-2* mutant *C*. *elegans* requires a fully functional BCL-11 to safeguard reproduction when conditions are unfavorable. As an alternative explanation, aberrant BCL-11 activity may interfere with DAF-16-dependent regulation of reproductive success, with negative consequence on embryonic development and brood size.

Furthermore, our current data also suggest that BCL-11 contributes to survival of IIS-deficient animals when adult animals are exposed to harsh environmental conditions. Indeed, we found that hypomorphic BCL-11 variants compromise the well-known tolerance of *daf-2* mutants to heat stress and high salinity. As expected, and in line with the current literature,^47^^,^^51^^,^^52^ *daf-16(lof)* could not rescue the aberrant phenotypes of adult *daf-2;bcl-11* mutants. At least for the experimental paradigms that we employed, our data indicate that BCL-11 may also be recruited in adult animals to fine-tune transcriptional programs that are relevant to stimulate cell resilience, thereby sustaining IIS-mediated cytoprotective effects in organisms exposed to environmental stress. As an added value, our study emphasizes once more that *(null)*/knockout alleles are not necessarily equivalent to disease-causing mutations, which may become fully penetrant and lead to heterogeneous phenotypes according to the organismal exposure to secondary challenges that weaken the system and push it beyond the limits of compensation. By employing hypomorphic variants and different experimental paradigms, we were able to reveal previously unknown functions of BCL-11 as a regulator of stress response in adult animals. We believe that our findings may hold potential implications that are relevant for understanding the complex etiology of NDDs. In support of our conclusions, epidemiological and clinical data suggest that additional prenatal and perinatal factors (e.g., maternal immune activation, diets, medications and infections) may prime organisms to NDDs.^40^ Similarly, early-life adversity (e.g., malnutrition, stress) seems to increase the risk of cognitive and psychiatric disorders, possibly because of an immune response that may act as a “second hit” to potentially vulnerable neurons with an epigenetic landscape already predisposed to neurodevelopmental defects.^53^^,^^54^ In line with this evidence from human and transgenic mouse model data, we suggest that cells carrying hypomorphic BCL-11 mutations may not be able to build up transcriptional profiles suitable to counteract environmental insults. If so, the consequent loss of homeostatic and/or metabolic processes may undermine neuronal differentiation, maintenance and remodeling, with obvious consequence at the organismal levels. Although we are aware that our work is based exclusively on *C*. *elegans*, we believe that our conclusions might also extend to higher model organisms and may help to elucidate how gene-by-environment interactions shape the complex etiology of NDDs.

### Limitations of the study

Our study has focused on hypomorphic BCL-11 variants and their impact on processes occurring during development and upon environmental stress. Although we have demonstrated a functional interplay between BCL-11/BCL11A and DAF-16/FOXO, we could not elucidate the downstream targets of these two transcription factors and how they mechanistically mediate embryonic development. Additionally, our work does not provide any validation experiment in mammalian systems, which would have further strengthened our conclusions and the pathophysiological relevance of our findings.

## Resource availability

### Lead contact

Further information and request of reagents should be directed to and will be fulfilled by the lead contact, Daniele Bano (daniele.bano@dzne.de).

### Materials availability

All *C*. *elegans* strains used in this study are fully available upon request. We may require a payment to cover shipping.

### Data and code availability

• All RNA sequencing raw data were deposited in GEO repository (GSE293058 and GSE293122).
• This article does not report original code.
• Any additional information required to reanalyze the data reported in this article are available from the lead contact upon request.

## Acknowledgments

We wish to express our gratitude to Ms. Christiane Bartling-Kirsch, Dr. Mrityunjoy Mondal, Ms. Ioanna-Maria Menegatou, and Mr. Michael Kraut for their support. B.X. was supported by a Chinese Scholarship Council (10.13039/501100002860CSC) fellowship (202108110050). This research was supported by the DZNE institutional budget. D.B. is a member of the Deutsche Forschungsgemeinschaft (10.13039/501100001659DFG, German Research Foundation) under Germany’s Excellence Strategy – EXC2151 – 390873048, Excellence Cluster ImmunoSensation^2^. D.B. and D.E. are members of the ETERNITY project consortium, funded by the European Union through Horizon Europe Marie Skłodowska-Curie Actions Doctoral Networks (MSCA-DN) under the grant number 101072759. S.B. was supported by the 10.13039/501100001659German Research Foundation (project-ID 227953431- SFB 1089) and the iBehave project funded from the program “Netzwerke 2021,” an initiative of the Ministry of Culture and Science of the State of North Rhine-Westphalia. The sole responsibility for the content of this publication lies with the authors. Some of the schemes were created with BioRender (license: Bano, D. [2025] https://BioRender.com/wczv7ej). Some strains were provided by the CGC, which is funded by the 10.13039/100000002NIH Office of Research Infrastructure Programs (P40 OD010440).

## Author contributions

P. Niedworok: investigation, formal analysis, and visualization; R.E.C.: investigation, formal analysis, and validation; B.X.: investigation, formal analysis, and validation; A.J.M.: investigation and formal analysis; B.J.: reagents; J.A.L.: reagents; E.D.D.: investigation and formal analysis; S.P.: investigation and formal analysis; M.B.: reagents and resources; P. Nicotera: funding acquisition; N.A.A.: investigation and formal analysis; D.E.: funding acquisition and resources; S.B.: conceptualization, formal analysis, writing – original draft, visualization, supervision, project administration, and funding acquisition; D.B.: conceptualization, formal analysis, writing – original draft, writing – review & editing, visualization, supervision, project administration, and funding acquisition.

## Declaration of interests

The authors declare no conflict of interest.

## Declaration of generative AI and AI-assisted technologies in the writing process

Text-generating AI tools (e.g., ChatGPT, Google Gemini) were used to improve the English syntax of a few sentences, in accordance with the journal’s guidelines. After using these tools, the authors reviewed and edited the content as needed and take full responsibility for the content of the publication.

## STAR★Methods

### Key resources table

**Table:** 

REAGENT or RESOURCE	SOURCE	IDENTIFIER
**Bacterial and virus strains**		

OP50 *E. coli,* B type	CGC	WBStrain00041969

**Chemicals, peptides, and recombinant proteins**		

Levamisole hydrochloride	Thermo Fisher Scientific	AC187870100
Agarose NEEO Ultra-Qualitat 1 kg	Carl Roth	2267.1
Sodium hydroxide	AppliChem	A4224
Sodium hypochlorite	VWR chemicals	27900.296
DNA ladder	New England BioLabs Inc.	NO467S 10161032
RedSafe	INtRON Biotechnology	21141
Proteinase K	PanReac AppliChem ITW Reagents	A4392
MyTag HS RNA Red Mix	Meridian Bioline	BIO-25048
TBE Buffer	PanReac AppliChem	A0972

**Deposited data**		

RNA sequencing raw data (relative to Figure 2)	This paper	GSE293058
RNA sequencing raw data (relative to Figure 3)	This paper	GSE293122

**Experimental models: Organisms/strains**		

*C. elegans* (hermaphrodites): wild type N2 (Bristol)	Caenorhabditis Genetics Center	WBStrain00000001
*C. elegans* (hermaphrodites): BAN1 *daf-2(e1370)*	Laboratory of Daniele Bano	https://doi.org/10.1016/j.celrep.2016.09.074
*C. elegans* (hermaphrodites): BAN155 *daf-16(mu86);daf-2(e1370)*	Laboratory of Daniele Bano	https://doi.org/10.1016/j.celrep.2016.09.074
*C. elegans* (hermaphrodites): BAN287 *daf-16(mu86);age-1(hx546)*	Laboratory of Daniele Bano	Reported in this paper
*C. elegans* (hermaphrodites): BAN713 *bcl-11(bon125)*	Laboratory of Daniele Bano	This paper
*C. elegans* (hermaphrodites): BAN728 *bcl-11(bon144)*	Laboratory of Daniele Bano	This paper
*C. elegans* (hermaphrodites): BAN742 *bcl-11(bon145)/nT1[umnIs49]*	Laboratory of Daniele Bano	This paper
*C. elegans* (hermaphrodites): BAN763 *daf-2(e1370*);*bcl-11(bon125)*	Laboratory of Daniele Bano	This paper
*C. elegans* (hermaphrodites): BAN764 *daf-2(e1370);bcl-11(bon144)*	Laboratory of Daniele Bano	This paper
*C. elegans* (hermaphrodites): BAN790 *daf-16(mu86);daf-2(e1370);bcl-11(bon125)*	Laboratory of Daniele Bano	This paper
*C. elegans* (hermaphrodites): BAN925 *bcl-11(bon172)/nT1[umnIs49]*	Laboratory of Daniele Bano	This paper
*C. elegans* (hermaphrodites): BAN929 *age-1(hx546);bcl-11(bon125)*	Laboratory of Daniele Bano	This paper
*C. elegans* (hermaphrodites): BAN930 *age-1(hx546);bcl-11(bon144)*	Laboratory of Daniele Bano	This paper
*C. elegans* (hermaphrodites): BAN956 *daf-16(mu86);age-1(hx546);bcl-11(bon125)*	Laboratory of Daniele Bano	This paper
*C. elegans* (hermaphrodites): BAN957 *daf-16(mu86);age-1(hx546);bcl-11(bon144)*	Laboratory of Daniele Bano	This paper
*C. elegans* (hermaphrodites): CB1370 *daf-2(e1370)*	Caenorhabditis Genetics Center	WBStrain00004309
*C. elegans* (hermaphrodites): CF1038 *daf-16(mu86)*	Caenorhabditis Genetics Center	WBStrain00004840

**Oligonucleotides**		

Primer: *bcl-11(bon125*) and *bcl-11(bon172)* Forward CGACGTGCTTCACCGGAAGA	This paper	N/A
Primer: *bcl-11(bon125*) and *bcl-11(bon172)* Reverse GGCGTAGTCTGGGACATCATATGGA	This paper	N/A
Primer: *bcl-11(bon144)* Forward GGGGGTTTCTGGTGTCGAATCAAC	This paper	N/A
Primer: *bcl-11(bon144)* Reverse CCTTCTCAATGAAGTTAGAGAAGCAAGAAAGG	This paper	N/A
Primer: *bcl-11(bon145)* Forward GTGAGCTTGCTGAGCCAGCT	This paper	N/A
Primer: *bcl-11(bon145)* Reverse CGGGTAAGAGAAGATTGAGCGCAA	This paper	N/A
Primer: *bcl-11 F_RT-PCR* CACATATTCCTGCGTGTTTCG	This paper	N/A
Primer: *bcl-11 R_RT-PCR* GAAGACGTTTCTATTGTCTGTG	This paper	N/A
Primer: *actin F_RT-PCR* GTGATGCCAGATCTTCTCCAT	This paper	N/A
Primer: *actin R_RT-PCR* GAGCACGGTATCGTCACCA	This paper	N/A

**Software and algorithms**		

R software	R Core Team	https://www.r-project.org
ImageJ	National Institutes of Health, Wayne Rasband	https://imagej.net/ij/
StepOne software	Thermo Fisher Scientific	https://www.thermofisher.com/de/de/home/technical-resources/software-downloads/StepOne-and-StepOnePlus-Real-Time-PCR-System.html
GraphPad Prism version 10	NSA	https://www.graphpad.com/features
BioRender	N/A	https://www.biorender.com

### Experimental model and study participant details

*C. elegans* was maintained in temperature-controlled incubators on nematode growth medium (NGM) plates seeded with B-type *Escherichia coli* strain OP50. The following strains were used: wild type N2 (Bristol), BAN1 *daf-2(e1370)*; BAN155 *daf-16(mu86);daf-2(e1370)*, BAN287 *daf-16(mu86);age-1(hx546)*, BAN713 *bcl-11(bon125)*, BAN728 *bcl-11(bon144)*, BAN742 *bcl-11(bon145)/nT1[umnIs49]*, BAN763 *daf-2(e1370);bcl-11(bon125)*, BAN764 *daf-2(e1370);bcl-11(bon144)*, BAN790 *daf-16(mu86);daf-2(e1370);bcl-11(bon125)*, BAN925 *bcl-11(bon172)/nT1[umnIs49]*, BAN929 *age-1(hx546);bcl-11(bon125)*, BAN930 *age-1(hx546);bcl-11(bon144)*, BAN956 *daf-16(mu86);age-1(hx546);bcl-11(bon125)*, BAN957 *daf-16(mu86);age-1(hx546);bcl-11(bon144)*, CB1370 *daf-2(e1370)*, CF1038 *daf-16(mu86)*. Gene editing was performed by using customized injection mixes consisting of a recombinant Cas9 and a sgRNAs targeting the coding sequence of the *bcl-11/F13H6.1* gene (InVivo Biosystems, Eugene OR, USA). Young adult animals were microinjected with a customized mix (plus a plasmid encoding *myo-2p::gfp* as a genetic reporter) and progeny were screened for GFP expression. GFP (+) nematodes were singled out and genotyped for the predicted mutation. Candidate *bcl-11* mutant lines were then backcrossed 3 to 6 times. For the genotyping of mutant nematodes, we used primer pairs as reported in key resources table.

### Method details

#### Bulk RNA-sequencing and analysis

RNA isolation was performed by using the RNeasy Micro kit (Qiagen), following manufacturer’s instruction. For the RNA sequencing, both the quantity and integrity of RNA were assessed using the HS RNA assay on a Tapestation 4200 system from Agilent. The Smart-seq2 protocol^55^ was employed to create non-strand-specific, full transcript sequencing libraries. In summary, 2 ng (adults) and 5 ng (eggs) of total RNA were transferred to a buffer containing 0.2% TritonX-100, protein-based RNase inhibitor, dNTPs, and oligo-dT oligonucleotides for priming the subsequent RT reaction on polyadenylated mRNA sequences. The SMART RT reaction was conducted at 42°C for 90 min using commercial SuperScript II (Invitrogen) and a Template-Switching Oligo. The pre-amplification PCR with 12 and 14 cycles respectively were carried out to produce double-stranded DNA from the cDNA template. Subsequently, 100 pg of amplified cDNA underwent tagmentation and enrichment using the Nextera XT kit (Illumina) to construct the final sequencing libraries. Quantification of libraries was performed using the Qubit HS dsDNA assay, and the distribution of library fragment sizes was determined using the D1000 assay on a Tapestation 4200 system (Agilent). The sequencing was executed in paired-end mode (75 cycles) on a NextSeq 2000 and a Novaseq X System (Illumina) with a NextSeq 2000 P4 XLEAP-SBS™ Reagent Kit (300 Cycles) and a Novaseq X Series 10B Reagent Kit (300 Cycle) chemistry respectively. Raw sequencing data were demultiplexed using bcl2fastq2 v2.20 and aligned against *C. elegans* reference genome (Ensembl 109) using Kallisto v048.0. All RNA sequencing raw data were deposited in GEO repository (GSE293058 and GSE293122).

#### Developmental assay

Synchronized nematodes were placed on individual NGM plates seeded with OP50 for 24h and larval stages were monitored and quantified until animals reached adulthood.

#### Egg-laying and hatching assays

For each individual experiment, at least 10 animals at L4 stage were singled out in 10 small OP50-containing plates. After 24h, adult nematodes were transferred onto new plates, while eggs were counted and incubated at either 20 or 25°C. Hermaphrodites were subsequently transferred to freshly seeded plates every 24h until the end of their reproductive cycle (approximately 8–10 days after hatching). The number of eggs laid and hatched (per animal) was also recorded every 24h until egg-laying ceased.

#### Hatching rate assay

Gravid adult animals at day 1 were individually placed on NGM plates seeded with *E. coli* OP50. After 4h of incubation at 20°C, adults were removed. Laid eggs were incubated at the indicated temperatures (20, 25, or 27°C). Egg hatching rates were quantified 48h later.

#### Lifespan assay

To perform lifespan assays, eggs were extracted from gravid young nematodes by using a hypochlorite solution. Eggs were then incubated at 20°C until animals reached L4 larval stage. For standard lifespan assay, L4 larvae were maintained at 20°C and adult animals were transferred every other day until all animals died. For experiments at 27°C, L4 larvae were transferred in new plates and then moved in a temperature-controlled incubator at 27°C until all animals died. Nematodes were scored every day by gentle touching, and those that did not respond were scored as dead. Censored nematodes were those that died of internal egg hatching, protruded vulvas or lost during transferring. Data were plotted using Kaplan-Meier survival curves and statistic was performed by using GraphPad Prism Software.

#### RNA extraction and quantitative real-time PCR

RNA was isolated from nematodes stored at −80°C with a QIAGEN RNeasy Kit according to the manufacturer’s instructions. Reverse transcription of the isolated RNAs was carried out using qscript cDNA Synthesis Kit (Quantabio), and RT-PCR was performed with SYBR green master mix (Applied Biosystems, USA) on an Applied Biosystems qRT-PCR Thermocycler. See key resources table for the primer pairs used in this study.

#### Stress assays

Heat stress assay: eggs were extracted from gravid young nematodes by using a hypochlorite solution. Around 50 animals (L4 larval stage) were singled-out in a plate and transferred to an incubator at 37°C. Nematodes were scored every 2h by gentle touching, and those that did not respond were scored as dead. The assay was carried out until all animals were dead. Osmotic stress assay: NGM plates with a final concentration of 500 mM NaCl were prepared. Around 50 nematodes (L4 larval stage) were transferred to a 500 mM NaCl plate for 24h at 20°C. Then, animals were moved to a standard NGM plate and after 24h they were scored by gentle touching.

#### Transcriptome- and phenome-wide association studies

We used TWAS-Fusion to assess the association of genetically proxied expression levels of *BCL11A* with BMI and type 2 diabetes based on GWAS summary statistics.^56^ Data on eQTLs were derived from the GTEx database, while GWAS summary statistics were derived from the combined meta-analysis of the GIANT and UK Biobank consortia for BMI, and from previously published studies (GWAS catalogue no.: GCST90018926).^57^^,^^58^ The gene-based phenome-wide association study was performed using the ExPheWas platform,^33^ which leverages data from the UK Biobank study to assess the association between variants in a particular gene and 362 self-reported diseases, 21 manually defined cardiovascular endpoints, 1280 phecodes and 83 standardized laboratory and anthropometric measurements, while adjusting for age, sex and the first 10 genetic principal components. For the phenome-wide association study a false discovery rate (FDR) correction was applied to account for multiple comparisons. The GTEx data used for the analyses described in this manuscript were obtained from dbGaP accession number phs000424.v10.p2 NHGRI GTEx on 07/29/2024.

### Quantification and statistical analysis

Statistical analyses were performed with GraphPad Prism Software. Statistical significance was determined using one-way ANOVA with Dunnett’s multiple comparisons test (Figure 1D), one-way ANOVA with Tukey’s multiple comparisons test (Figures 3A, 3B, 3D–3G, 4A, and 4B), and one-way ANOVA with Šidák’s multiple comparisons test (Figure 3C). Biological replicates are indicated as n and are reported in the figure legends. For lifespan analyses, statistical significance was determined using a Mantel-Cox (log rank) test in GraphPad Prism (10.6.1) (see Table S1). For the remaining experiments, details about the statistical analyses are indicated in figure legends.

## Supplemental information

Table S1. Supplementary table reports genotypes, number of animals (dead and censored events), median and maximum lifespan (days) for each invidividual experiment (Exp) performed in this study

Table S2. ExPheWas summary of the gene-based phenome-wide association study results (only outcomes with FDR Q < 0.05 are displayed)
