# Supplementary material for: BCL-11 enables adaptive stress responses to environmental challenges
Source: iScience. 2026 Jan 7;29(2):114422. doi: 10.1016/j.isci.2025.114422 (PMC12865584; doi:10.1016/j.isci.2025.114422)
Supplement: Table S1. Supplementary table reports genotypes, number of animals (dead and censored events), median and maximum lifespan (days) for each invidividual experiment (Exp) performed in this study [file mmc1.pdf]

iScience, Volume *29*

Supplemental information

BCL-11 enables adaptive stress

responses to environmental challenges

Patrizia Niedworok, Rossella Erminia Ciliberti, Beijia Xie, Amal John Mathew, Benjamin Jussila, Jennifer A. Lawson, Elena De Domenico, Stefan Paulusch, Marc Beyer, Pierluigi Nicotera, N. Ahmad Aziz, Dan Ehninger, Sandra Blaess, and Daniele Bano

Supplementary table ST1.

Supplementary table reports genotypes, number of animals (dead and censored events), median and maximum lifespan (days) for each invidividual experiment (Exp) performed in this study. Log-rank (Mantel-Cox) test was used to calculate *p* values (relative to one or multiple genotypes: see below).

**Lifespan assay (20°C, from hatching)**					
**Exp**	**Genotypes**	**Dead (Censored)**	**Median (days)**	**Max (days)**	***p value***
1	wt (N2)	105(25)	24	29	-
	bcl-11(bon125)	117(18)	23	31	0.0292[a]
	bcl-11(bon144)	103(29)	25	31	0.0013[a]
	daf-2(e1370)	110(29)	54	71	<0.0001[a]
	daf-2(e1370);bcl-11(bon125)	71(17)	53	70	<0.0001[a]<br>0.1170[b]
	daf-2(e1370);bcl-11(bon144)	117(22)	54	69	<0.0001[a]<br>0.0537[b]
	daf-16(mu86);daf-2(e1370)	93(41)	23	29	0.0019[a]<br><0.0001[b]
	daf-16(mu86);daf-2(e1370);bcl-11(bon125)	120(30)	23	29	0.0112[a]<br><0.0001[b]<br>0.4302[c]
2	wt (N2)	113(22)	24	33	-
	bcl-11(bon125)	107(10)	24	33	0.1415[a]
	bcl-11(bon144)	115(12)	25	38	0.1785[a]
	daf-2(e1370)	88(46)	53	66	<0.0001[a]
	daf-2(e1370);bcl-11(bon125)	121(39)	53	68	<0.0001[a]<br>0.7376[b]
	daf-2(e1370);bcl-11(bon144)	113(46)	53	66	<0.0001[a]<br>0.1739[b]
	daf-16(mu86);daf-2(e1370)	100(37)	23	26	<0.0001[a]<br><0.0001[b]
	daf-16(mu86);daf-2(e1370);bcl-11(bon125)	114(43)	23	26	<0.0001[a]<br><0.0001[b]<br>0.0514[c]
3	wt (N2)	97(16)	26	33	-
	bcl-11(bon125)	94(10)	25	34	0.2755[a]
	bcl-11(bon144)	98(19)	24	34	0.0426[a]
	daf-2(e1370)	99(18)	55	70	<0.0001[a]
	daf-2(e1370);bcl-11(bon125)	99(12)	53	69	<0.0001[a]<br>0.5987[b]
	daf-2(e1370);bcl-11(bon144)	102(27)	54	69	<0.0001[a]<br>0.8528[b]
	daf-16(mu86);daf-2(e1370)	94(41)	23	28	<0.0001[a]<br><0.0001[b]
	daf-16(mu86);daf-2(e1370);bcl-11(bon125)	99(24)	23	28	<0.0001[a]<br><0.0001[b]<br>0.1773[c]

[a] *p* value compared to wt (N2)
[b] *p* value compared to *daf-2(e1370)*
[c] *p* value compared to *daf-16(mu86);daf-2(e1370)*

Lifespan assay (hatching at 20°C, then L4 transferred to 27°C)					
**Exp**	**Genotypes**	**Dead (Censored)**	**Median (days)**	**Max (days)**	***p value***
4	wt (N2)	85(18)	12	18	-
	daf-2(e1370)	95(2)	39	55	<0.0001[a]
	daf-2(e1370);bcl-11(bon125)	95(2)	35	46	<0.0001[a] 0.0058[b]
	daf-2(e1370);bcl-11(bon144)	108(1)	31	42	<0.0001[a] <0.0001[b]
5	wt (N2)	109(4)	13	16	-
	daf-2(e1370)	98(15)	36	50	<0.0001[a]
	daf-2(e1370);bcl-11(bon125)	96(5)	36	48	<0.0001[a] 0.9111[b]
	daf-2(e1370);bcl-11(bon144)	93(7)	32	44	<0.0001[a] <0.0001[b]
6	wt (N2)	87(9)	13	17	-
	daf-2(e1370)	97(4)	36	58	<0.0001[a]
	daf-2(e1370);bcl-11(bon125)	107(1)	36	45	<0.0001[a] 0.1929[b]
	daf-2(e1370);bcl-11(bon144)	98(1)	32	43	<0.0001[a] <0.0001[b]
7	wt (N2)	81(13)	13	22	-
	bcl-11(bon125)	105(9)	13	20	0.4568[a]
	bcl-11(bon144)	96(11)	13	19	0.1672[a]
8	wt (N2)	119(3)	13	20	-
	bcl-11(bon125)	107(6)	13	20	0.0120[a]
	bcl-11(bon144)	107(6)	13	20	0.3936[a]
9	wt (N2)	91(5)	11	17	-
	bcl-11(bon125)	79(13)	11	15	0.0871[a]
	bcl-11(bon144)	79(10)	11	17	0.6478[a]

[a]*p* value compared to wt (N2)
[b]*p* value compared to *daf-2(e1370)*
