# Supplementary material for: BCL-11 enables adaptive stress responses to environmental challenges
Source: iScience. 2026 Jan 7;29(2):114422. doi: 10.1016/j.isci.2025.114422 (PMC12865584; doi:10.1016/j.isci.2025.114422)
Supplement: Table S2. ExPheWas summary of the gene-based phenome-wide association study results (only outcomes with FDR Q < 0.05 are displayed) [file mmc2.pdf]

Supplementary table ST2.

ExPheWas summary of the gene-based phenome-wide association study results (only outcomes with FDR Q < 0.05 are displayed)

Outcome Id	Description	N	-log10(P)	P-Value	P-Value (Bonferroni)	Q-Value
cont_v27	Mean corpuscular volume	401,819	123.98	1.00E-124	1.70E-121	1.40E-121
cont_v28	Mean corpuscular haemoglobin	401,813	98.96	1.10E-99	1.80E-96	7.30E-97
cont_v49	Mean reticulocyte volume	395,457	81.73	1.90E-82	3.00E-79	8.30E-80
cont_v24	Red blood cell (erythrocyte) count	401,820	64.13	7.40E-65	1.20E-61	2.50E-62
cont_v50	Mean sphered cell volume	395,235	63.13	7.40E-64	1.20E-60	2.00E-61
cont_v30	Red blood cell (erythrocyte) distribution width	401,817	40.63	2.30E-41	3.80E-38	5.20E-39
cont_v69	Glycated haemoglobin (HbA1c)	395,008	17.05	8.80E-18	1.40E-14	1.70E-15
cont_v51	Immature reticulocyte fraction	395,234	9.72	1.90E-10	3.10E-07	3.20E-08
cont_v5	Body mass index (baseline)	411,767	7.41	3.90E-08	6.30E-05	5.70E-06
cont_v66	Cystatin C	394,944	7.29	5.10E-08	8.30E-05	6.80E-06
cont_v53	High light scatter reticulocyte count	395,234	6.14	7.20E-07	0.001	7.40E-05
cont_v3	Body fat percentage (baseline)	405,649	5.96	1.10E-06	0.002	1.00E-04
cont_v48	Reticulocyte count	395,457	5.65	2.20E-06	0.004	2.00E-04
cont_v82	Urate	394,508	5.15	7.10E-06	0.011	5.90E-04
cont_v15	Forced expiratory volume in 1-second (FEV1), Best measure	310,594	4.86	1.40E-05	0.023	0.001
cont_v4	Forced expiratory volume in 1 second (FEV1)	310,594	4.86	1.40E-05	0.023	0.001
cont_v25	Haemoglobin concentration	401,819	4.38	4.10E-05	0.067	0.003
cont_v52	High light scatter reticulocyte percentage	395,235	4.27	5.40E-05	0.088	0.003
cont_v8	Waist circumference	412,401	4.07	8.50E-05	0.138	0.005
cont_v16	Forced vital capacity (FVC), Best measure	310,594	3.89	1.30E-04	0.212	0.007
cont_v26	Haematocrit percentage	401,820	3.67	2.20E-04	0.351	0.011
cont_v11	Forced vital capacity (FVC)	310,594	3.6	2.50E-04	0.41	0.012
cont_v7	Basal metabolic rate (baseline)	405,857	3.52	3.00E-04	0.494	0.014
cont_v70	HDL cholesterol	362,463	3.39	4.10E-04	0.665	0.018
cont_v57	Apolipoprotein A	360,446	3.37	4.30E-04	0.694	0.018
cont_v19	Forced expiratory volume in 1-second (FEV1) Z-score	331,069	3.14	7.20E-04	1	0.03
cont_v55	Alkaline phosphatase	394,999	3.12	7.60E-04	1	0.031
cont_v21	FEV1/ FVC ratio Z-score	331,069	3.1	7.90E-04	1	0.031
cont_v37	Neutrophill count	401,122	2.93	0.001	1	0.042
cont_v40	Nucleated red blood cell count	400,925	2.89	0.001	1	0.045

ExPheWas summary of the gene-based phenome-wide association study results (only outcomes with FDR Q < 0.05 are displayed)

Outcome Id	Description	N cases	N controls	N excluded from controls	-log10(P)	P-Value	P-Value (Bonferroni)	Q-Value
250.2	Type 2 diabetes	30,704	380,299	2,130	4.59	2.60E-05	0.042	0.002
418.1	Precordial pain	6,573	370,862	35,698	3.45	3.60E-04	0.582	0.016
574.3	Cholecystitis without cholelithiasis	3,950	387,346	21,837	3.01	9.90E-04	1	0.037
411.8	Other chronic ischemic heart disease, unspecified	25,224	363,761	24,148	3.01	9.90E-04	1	0.037
